# HIV‐1 viral load and reservoir size remain stable following SARS‐CoV‐2 mRNA vaccination in people with HIV

**DOI:** 10.1111/hiv.70235

**Published:** 2026-04-05

**Authors:** Oscar Kieri, Bianca B Jütte, Jan Vesterbacka, Soo Aleman, Hans‐Gustaf Ljunggren, Marcus Buggert, J Peter Svensson, Anders Sönnerborg, Piotr Nowak

**Affiliations:** ^1^ Department of Infectious Diseases Karolinska University Hospital Stockholm Sweden; ^2^ Department of Medicine Huddinge CIM ‐ Clinical Infectious Diseases, Karolinska Institutet Stockholm Sweden; ^3^ Department of Medicine Huddinge Center for Infectious Medicine, Karolinska Institutet Stockholm Sweden; ^4^ Department of Translational Pharmacology Bielefeld University, Medical School OWL Bielefeld Germany; ^5^ Department of Clinical Microbiology Karolinska University Hospital Stockholm Sweden

**Keywords:** HIV‐1 reservoir, immunological non‐responders, SARS‐CoV‐2 mRNA vaccination, viral load

## Abstract

**Background:**

The potent immunogenicity of mRNA vaccination raised concerns about its impact on HIV‐1 viral load (VL) and reservoir size in people with HIV, which could drive vaccine hesitancy. As part of a prospective clinical trial, we investigated whether SARS‐CoV‐2 mRNA vaccination induced changes in VL and reservoir size after a 1‐year three‐dose regimen in people with HIV on antiretroviral therapy (ART).

**Methods:**

We collected blood samples from 37 people with HIV with varying degrees of immune reconstitution before vaccination and after receiving their second and third doses of vaccine. Anti‐SARS‐CoV‐2‐Spike antibodies and Spike‐specific CD4^+^T‐cells were analysed as indicators of vaccine‐induced immunogenicity. The Intact Proviral DNA Assay was used to quantify the intact and defective HIV‐1 reservoir.

**Results:**

Vaccination with three doses of SARS‐CoV‐2 mRNA vaccine over the course of 1 year did not result in plasma HIV‐1 RNA rebound nor significantly alter the HIV‐1 reservoir size regardless of immune status. The median intact HIV‐1 reservoir size was 53 (IQR 34–193) copies/million CD4^+^T‐cells at baseline and 86 (34–268) and 138 (33–334) 3 months after the second and third dose, respectively (*p* > 0.38). We found no correlation between changes in VL or HIV‐1 reservoir size and the anti‐SARS‐CoV‐2‐Spike levels, Spike‐specific CD4^+^T‐cells, CD4^+^/CD8^+^ ratio, nadir or CD4^+^T‐cell count. However, the intact reservoir size negatively correlated with CD4^+^T‐cell count, CD4^+^/CD8^+^ ratio and nadir CD4^+^T‐cell count.

**Conclusions:**

Our findings show that three doses of SARS‐CoV‐2 mRNA vaccine did not affect the VL or HIV reservoir size regardless of immune status, supporting their safety in people with HIV. Notably, a higher CD4^+^T‐cell count correlated with a reduced intact HIV‐1 reservoir size.

## BACKGROUND

The high immunogenicity of mRNA vaccines raised concerns about the impact of SARS‐CoV‐2 mRNA vaccines on HIV‐1 viral rebound and reservoir size in people with HIV. At the same time, the widespread distribution of safe and effective SARS‐CoV‐2 mRNA vaccines played a pivotal role in addressing the challenges posed by the COVID‐19 pandemic. Recognizing that people with HIV faced an elevated risk of severe COVID‐19 outcomes early in the pandemic, prioritizing vaccination for this group among other immunocompromised groups became an important objective [1, 2, 3].

There is now evidence that people with HIV under suppressive antiretroviral therapy (ART) with adequate immune reconstitution generally exhibit a robust immune response to COVID‐19 vaccination [4, 5, 6, 7]. Moreover, the risk of severe COVID‐19 outcomes after two and especially after three doses of vaccine is comparable to that of individuals without HIV‐1 [8, 9, 10]. Still, people with HIV constitute a heterogeneous group with varying levels of immune deficiency and viral control, which may influence vaccine response and clinical outcomes. Even on ART, the degree and rate of immune reconstitution vary greatly among individuals. Despite sustained virological suppression, CD4^+^ T‐cell counts fail to normalize in up to 30% of people with HIV [11]. Immunological non‐responders (INRs), with a CD4^+^ T‐cell count below 350 cells/mm^3^, and individuals with elevated viral load (VL) are still at greater risk of adverse outcomes and poorer response to vaccination when compared to immunological responders (IRs) and aviraemic individuals [3, 6, 12, 13, 14, 15]. Furthermore, people with HIV under successful ART continue to experience chronic immune activation and inflammation, which may influence the vaccine response and immunological effects [16].

Vaccines have had a profound impact on society and human survival ever since the first success story of cowpox exposure and protection against smallpox in the 18th century. The benefits outweigh the risks in well‐designed vaccines, but the necessary immune response generated from the vaccine could cause temporary inflammation, an influence that remains a common driver of vaccine hesitancy [17]. Even if earlier studies have shown that the vaccine‐mediated immune response following vaccination against influenza, hepatitis B and pneumococcus could stimulate viral rebound in people with HIV, the effects on the viral reservoir remain unclear [18, 19, 20]. Furthermore, mRNA vaccines, which are potent stimulators of the adaptive immune response involving cells that harbour proviral HIV DNA, could potentially stimulate viral rebound. Studies have shown that INRs have a larger proviral reservoir [21, 22] and increased inflammation and morbidity [23, 24]. Hypothetically, mRNA vaccines could have a greater effect on the proviral reservoir in this group of individuals. Existing studies of SARS‐CoV‐2 mRNA vaccines in people with HIV have shown both inducible viraemia and absence of effect on viral rebound. The impact on viral reservoirs has been limited. Overall, studies have been performed predominantly on men with high CD4^+^ T‐cell levels and diverse vaccines with limited follow‐up time after two doses of vaccine [25, 26, 27]. To address these knowledge gaps, we explored the impact of three doses of SARS‐CoV‐2 mRNA vaccine over one year in a diverse population with varying immune status, assessing its potential effect on plasma HIV‐1 VL and HIV‐1 reservoir size.

## METHODS

Individuals included in this study were part of the COVAXID trial, an open‐label, non‐randomized prospective clinical trial at the Karolinska University Hospital, Stockholm, Sweden, to investigate the safety and clinical efficacy of the mRNA BNT162b2 vaccine (Comirnaty®, Pfizer/BioNTech) in immunocompromised individuals [4, 5].

### Patient consent statement

Ethical approval was obtained from the Swedish Ethical Review Authority (ID 2021‐00451, ID 2023‐05153‐02), and all participants provided written informed consent. All methods of this study were performed in accordance with the Declaration of Helsinki and Good Clinical Practice guidelines. The trial was registered at the European Union Drug Regulating Authorities Clinical Trials Database (EudraCT 2021‐000175‐37) and clinicaltrials.gov (NCT04780659) by Feb 9, 2021, and Feb 19, 2021, respectively. The trial was approved by the Swedish Medical Product Agency (ID 5.1‐2021‐5881).

### Study cohort

People with HIV aged 18–85 years who were followed at the outpatient HIV clinic at the Karolinska University Hospital and were eligible for COVID‐19 vaccination, with no known history of SARS‐CoV‐2 infection, were screened for inclusion in the trial. Recruitment started on Feb 15, 2021, initial follow‐up ended Oct 15, 2021, and the trial was fully recruited as intended in the study plan. The clinical trial was subsequently extended for a period of up to 2 years. Ninety people with HIV on effective ART were included in the clinical trial, and 50 of the participants, in subgroups of IR (CD4 ≥ 350) and INR (CD4 < 350), were sampled for HIV‐1 reservoir analysis. After a follow‐up of 1 year, post three doses of mRNA vaccine, 37 participants had a successful reservoir analysis for baseline and at least one additional time point—these are described in this study.

The two initial vaccine doses, mRNA BNT162b2 (Pfizer/BioNTech), were administered 3 weeks apart (day 0 and day 21). The third dose, mRNA BNT162b2 (Pfizer/BioNTech) or mRNA‐1273 (Moderna), was administered on average 9 months following the second dose. Samples for VL and anti‐SARS‐CoV‐2‐Spike antibodies were collected at baseline (day 0) and at seven timepoints until 3 months after the third dose of mRNA vaccine (days 10, 21, 35 and months 3, 6, 9 and 12). Samples for Spike‐specific CD4^+^ T‐cells were collected 2 weeks after the second dose (day 35). Blood samples for reservoir analysis were collected prior to vaccination on the day of the first vaccine dose (day 0), and 3 months after the second and third vaccine doses. Clinical characteristics were extracted from InfCareHIV, the Swedish national quality registry for HIV and electronic health records.

### Immunological and virological measurements

CD4^+^ and CD8^+^ T‐cell counts, and HIV‐1 VL were determined by flow cytometry and Cobas Amplicor (Roche Molecular Systems Inc., USA), respectively. SARS‐CoV‐2 spike IgG assays were performed in serum (Elecsys Anti‐SARS‐CoV‐2 S; Roche Diagnostics International Ltd., Switzerland). Dilution of serum up to 1/100 was done when needed, allowing measurement of IgG levels up to 25 000 U/mL, in contrast to using the standard upper limit level of 250 U/mL [4]. Spike‐specific CD4^+^ T‐cell responses were quantified using activation‐induced marker assays via up‐regulation of CD69 and CD40L (CD154), as previously described [28]. SARS‐CoV‐2 IgG nucleocapsid antibodies were analysed using V‐PLEX SARS‐CoV‐2 (Meso Scale Diagnostics, MSD).

### Reservoir analysis

We performed the Intact Proviral DNA Assay (IPDA) to estimate the intact and defective HIV‐1 reservoir [29]. Peripheral blood mononuclear cells (PBMCs) were isolated by density gradient separation, and resting CD4^+^ T‐cells (rCD4^+^ T‐cells) were isolated from the obtained PBMCs by a two‐step magnetic isolation using the Miltenyi Biotec magnetic associated cell sorting (MACS) platform. For the first step, the CD4 negative cell isolation kit (Miltenyi Biotec, Cat# 130–096‐533) was used according to the manufacturer's protocol. Secondly, resting CD4^+^ T‐cells were negatively selected with magnetic beads against the activation markers CD25, CD69 and HLA‐DR. DNA was extracted by QIAamp DNA Mini Kit (Qiagen, Cat#51304); a median of 1.6 (IQR 1.0, 1.9) ug DNA was extracted per sample.

For IPDA, digital droplet PCR (ddPCR) was performed with the QX200 Droplet Digital qPCR System (Bio‐Rad). Each reaction consisted of 20 μL, containing 10‐μL Supermix for Probes without dUTP (Bio‐Rad, Cat# 1863024), 900‐nM primers, 250‐nM probe (labelled with HEX or FAM) and 8‐μL undiluted cellular DNA. Blood from individuals without HIV (*n* = 4) were used in parallel as negative controls samples. As a positive control we used a J‐Lat cell lines 5A8 [30, 31]. Droplets were generated using the QX200 droplet generator. Emulsified PCR reactions were performed with a C1000 Touch thermal cycler (Bio‐Rad), with the following protocol: 95 °C for 10 min, followed by 40 cycles of 94 °C for 30 s and 57 °C for 60 s, and a final droplet cure step of 10 min at 98 °C. Each well was then read with a QX200 Droplet Reader (Bio‐Rad). Droplets were analysed with QuantaSoft version 1.5 (Bio‐Rad) software in the absolute quantification mode.

### Statistical analysis

Comparisons of continuous variables between groups were conducted using the Mann–Whitney U test for unpaired data, the Wilcoxon signed‐rank test for paired data and repeated‐measures analysis of variance (ANOVA) for paired data over multiple time points. Correlations between continuous variables were assessed using Spearman's correlation, while frequency comparisons were analysed using Fisher's exact test. Statistical analyses were performed using GraphPad Prism version 10.4.1 (532) for Mac, GraphPad Software.

## RESULTS

### Study participants

The study included 37 participants with a median age of 54 years, of whom 41% were female (Table 1). All participants were receiving ART at study inclusion (median 7 years), and 81% were treated with integrase strand transfer inhibitors. At baseline, prior to the first vaccine dose, the median CD4^+^ T‐cell count was 500 cells/mm^3^ (IQR 280–685), and 73% and 89% of participants had a VL of <20 and <50 copies/ml (maximum: 77 copies/ml), respectively. In total, 54% had a nadir CD4^+^ T‐cell count less than 200 cells/mm^3^, and the median nadir CD4^+^ T‐cell count was 180 cells/mm^3^ (IQR 65–385).

**TABLE 1:** Baseline characteristics.

Characteristic	All (*n* = 37)^a^	IR (*n* = 22)^a^	INR (*n* = 15)^a^	*p* value^b^
Age at enrolment	54 (43, 66)	54 (42, 64)	56 (44, 69)	0.70
Sex at birth				0.51
Female	15 (41)	10 (45)	5 (33)	
Male	22 (59)	12 (55)	10 (67)	
CD4^+^ T‐cell count (cells/mm^3^)	500 (280, 685)	660 (578, 740)	280 (200, 290)	<0.001
CD4^+^/CD8^+^ ratio	0.86 (0.44, 1.3)	1.29 (0.94, 1.73)	0.42 (0.31, 0.55)	<0.001
Nadir CD4^+^ T‐cell count (cells/mm^3^)	180 (65, 385)	345 (225, 455)	40 (10, 140)	<0.001
HIV‐1 RNA <20 copies/mL	27 (73)	18 (82)	9 (60)	0.14
Years since HIV diagnosis	8 (4, 20)	13 (7, 21)	4 (3, 11)	0.02
Years on ART	7 (4, 15)	10 (6, 19)	4 (3, 11)	0.02
Primary HIV infection	2 (5)	2 (9)		0.50
Current ART regimen				0.81
INSTI‐based	30 (81)	18 (82)	12 (80)	
NNRTI‐based	6 (16)	3 (14)	3 (20)	
PI‐based	1 (3)	1 (4)		
Route of transmission				0.57
Heterosexual	25 (67)	16 (73)	9 (60)	
MSM	11 (30)	6 (27)	5 (33)	
IV drug use	1 (3)		1 (7)	
Ethnicity				0.35
Asian	8 (22)	5 (23)	3 (20)	
Black	7 (19)	2 (9)	5 (33)	
Caucasian	19 (51)	13 (59)	6 (40)	
Latino	3 (8)	2 (9)	1 (7)	
COVID‐19 naïve throughout follow‐up	27 (73)	16 (73)	11 (73)	1.00
SARS‐CoV‐2 mRNA vaccine regimen				
First and second dose				
BNT162b2	37 (100)	22 (100)	15 (100)	
Third dose				0.73
BNT162b2	24 (65)	15 (68)	9 (60)	
mRNA‐1273	13 (35)	7 (32)	6 (40)	

Abbreviations: ART, antiretroviral therapy; INSTI, integrase strand transfer inhibitor; MSM, men who have sex with men; NNRTI, non‐nucleoside analogue reverse transcriptase inhibitor; PI, protease inhibitor.

^a^ Median (Q1, Q3); *n* (%).

^b^ Mann–Whitney *U* test; Fisher's exact test.

Among INRs (*n* = 15), the median CD4^+^/CD8^+^ ratio, CD4^+^ T‐cell count and nadir were 0.42, 280 cells/mm^3^ and 40 cells/mm^3^, respectively. Among IRs (*n* = 22), the median CD4^+^/CD8^+^ ratio, CD4^+^ T‐cell count and nadir were 1.29, 660 cells/mm^3^ and 345 cells/mm^3^, respectively. Further characteristics of the total cohort, stratified into the groups of INR and IR, are presented in Table 1. The demographic and clinical characteristics of the study cohort were comparable to those of the overall population of people with HIV included in the COVAXID trial, as reported in the primary trial publication [4].

All participants received the BNT162b2 (Pfizer/BioNTech) as the first and second vaccine doses, administered 3 weeks apart. The third dose, administered on average 9 months after baseline, consisted of BNT162b2 in 65% of participants and mRNA‐1273 (Moderna) in 35%. During follow‐up, COVID‐19 infection in participants did occur; however, 73% remained COVID‐19 naïve throughout follow‐up, confirmed by negative SARS‐CoV‐2 Nucleocapsid IgG Antibody results.

### HIV‐1 RNA VL following vaccination

**FIGURE 1:**
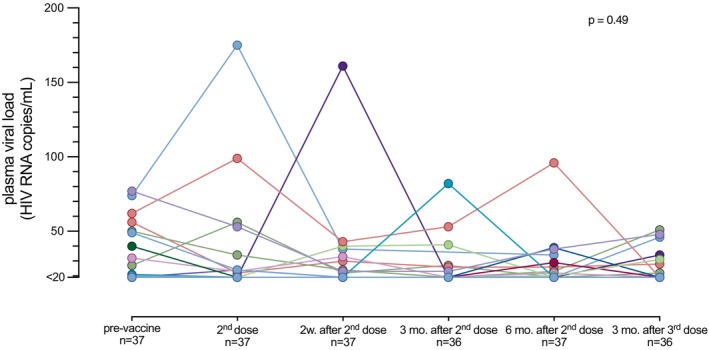
Plasma HIV‐1 RNA dynamics following SARS‐CoV‐2 mRNA vaccination. Plasma HIV‐1 RNA levels measured pre‐vaccination (on the same day prior to the first dose of vaccine); on the day of the second dose; and at 2 weeks, 3 months and 6 months after the second dose, as well as 3 months after the third dose. Each line represents an individual participant. Lower limit of quantification (LLOQ) 20 copies/mL. Mean HIV‐1 RNA across timepoints: 27, 28, 26, 23, 23 and 22 copies/mL, respectively. No significant changes in HIV‐1 RNA were observed (repeated‐measures one‐way ANOVA, *p* = 0.49).

To assess whether the vaccination induced viraemia, HIV‐1 RNA was measured on the day before the first dose of vaccine, on the day of the second dose, and 2 weeks, 3 months and 6 months after the second dose, as well as 3 months after the third dose. We could not observe significant differences in HIV‐1 RNA VL before and after vaccination throughout the study (*p* = 0.49) (Figure 1). Prior to vaccination, 73% of participants had a VL of <20 copies/ml. The proportion of virally suppressed individuals increased to 78% 2 weeks after the second dose, to 83% 3 months after the second dose, and to 81% 3 months after the third dose (*p* = 0.75). Similarly, no significant differences were observed in mean HIV‐1 RNA level or in the proportion of virally suppressed individuals when comparing IRs and INRs throughout follow‐up (all *p* > 0.42).

### HIV‐1 reservoir size following vaccination

**FIGURE 2:**
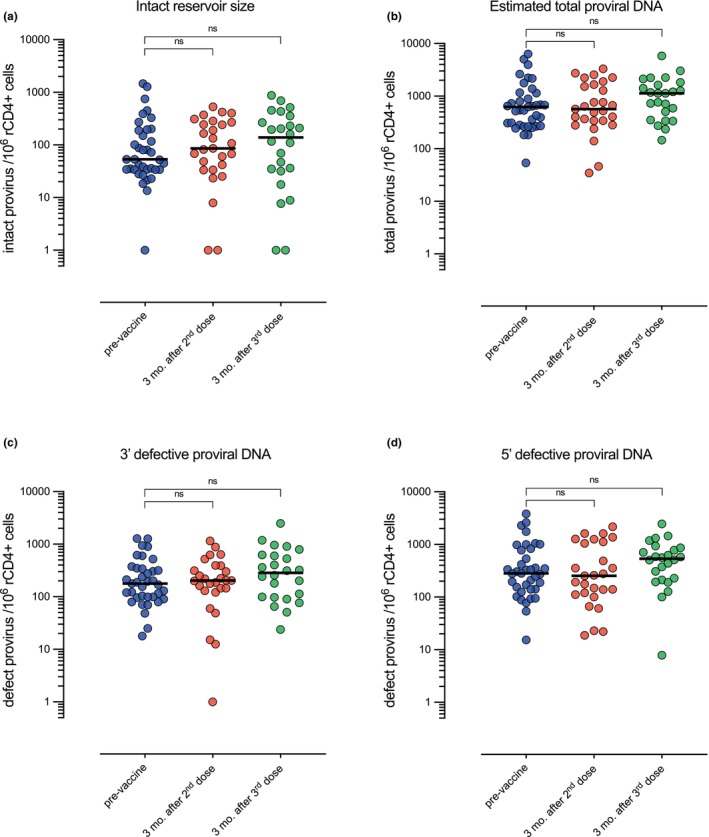
Longitudinal dynamics of the HIV‐1 proviral reservoir following SARS‐CoV‐2 mRNA vaccination. (a) Intact HIV‐1 reservoir, (b) total proviral HIV‐1 DNA, (c) 3′ defective and (d) 5′ defective proviral DNA (copies/million resting CD4^+^ T‐cells) quantified using the Intact Proviral DNA Assay (IPDA) at baseline (pre‐vaccine, day 0, blue, *n* = 37), 3 months after two vaccine doses (red, *n* = 27) and 3 months after three doses (green, *n* = 24). Points represent individual values, and horizontal bars represent median values. No significant changes in reservoir size were observed following vaccination (Wilcoxon signed‐rank and Mann–Whitney U tests). Baseline versus 3 months after the second dose, *p* = 0.09–0.88 (Wilcoxon), 0.47–0.85 (Mann–Whitney). Baseline versus 3 months after the third dose, *p* = 0.19–0.41 (Wilcoxon), 0.10–0.39 (Mann–Whitney).

To evaluate changes in the proviral reservoir size following vaccination, we quantified the number of intact and defective HIV‐1 DNA copies per million resting CD4^+^ T‐cells in blood using IPDA (Figure 2). We observed no significant changes in the intact, defective or total proviral reservoir following two and three doses of SARS‐CoV‐2 mRNA vaccine. At baseline, prior to vaccination, the median intact HIV‐1 reservoir size was 53 copies/million rCD4^+^ T‐cells (IQR 34–193). Three months after the second vaccine dose, the median intact reservoir size was 86 copies/million rCD4^+^ T‐cells (IQR 34–268) (Wilcoxon signed‐rank test *p* = 0.09; Mann–Whitney U test *p* = 0.47), and three months after the third dose, it was 138 copies/million rCD4^+^ T‐cells (IQR 33–334) (Wilcoxon *p* = 0.36; Mann–Whitney *p* = 0.39), confirming no significant changes following vaccination (Figure 2a). Similarly, the 3′ defective, 5′ defective, and estimated total proviral reservoir did not change significantly after two or three doses of vaccine (Wilcoxon, all *p* > 0.19) (Figure 2b–d).

### HIV‐1 RNA VL in correlation to vaccine‐induced immune response

**FIGURE 3:**
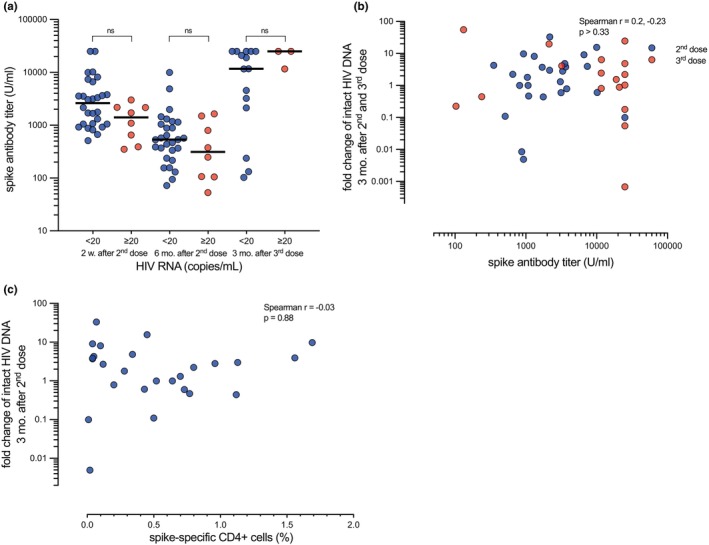
SARS‐CoV‐2 mRNA vaccine‐induced immune response in relation to HIV‐1 RNA and fold change of intact HIV‐1 reservoir. (a) SARS‐CoV‐2‐Spike antibody titre (U/mL) in people with HIV stratified by HIV‐1 RNA <20 (LLOQ) (blue) and ≥ 20 copies/mL (red), 2 weeks after second vaccine dose (*n* = 36), 6 months after second dose (*n* = 36) and 3 months after third dose (*n* = 18) of SARS‐CoV‐2 mRNA vaccine (Mann–Whitney U test, *p* = 0.07, 0.4, 0.29). (b) Correlation between fold change of intact HIV‐1 DNA (IPDA) from baseline (day 0) to 3 months after second dose (blue) or 3 months after third dose (red) and vaccine‐induced SARS‐CoV‐2‐Spike antibody titres (Spearman's correlation). (c) Correlation between fold change of intact HIV‐1 DNA from baseline to 3 months after second dose and the proportion of Spike‐specific CD4^+^ T‐cells (Spearman's correlation).

Participants in our study exhibited an overall robust immune response to vaccination, with anti‐SARS‐CoV‐2 Spike antibody titres comparable to those of the control group in the clinical trial [4]. To identify if the magnitude of the immune response could be linked to viraemia, we analysed the anti‐SARS‐CoV‐2 Spike titre in relation to HIV‐1 RNA levels 2 weeks and 6 months after the second vaccine dose, and 3 months after the third dose. Overall, Spike antibody titres did not differ significantly between individuals with HIV‐1 RNA <20 copies/ml and those with HIV‐1 RNA ≥20 copies/ml (Figure 3a). Two weeks after the second dose, there was a trend towards higher Spike antibody titres in virally suppressed individuals, with a median titre of 2612 U/mL (IQR 1049–5922) compared to 1406 U/mL (IQR 458–2181) in individuals with HIV‐1 RNA ≥20 copies/ml (*p* = 0.07). Six months after the second dose and 3 months after the third dose, median Spike antibody titres were 534 U/mL (IQR 262–1122) and 11 676 U/mL (IQR 2127–25 000), respectively, in individuals with HIV‐1 RNA <20 copies/ml, compared with 313 U/mL (IQR 106–1322) and 25 000 U/mL (IQR 11613–25 000) in those with ≥20 copies/ml (*p* = 0.4, *p* = 0.29).

### HIV‐1 reservoir size change in correlation to vaccine‐induced immune response and immune status

Likewise, to assess whether the magnitude of the vaccine‐induced immune response was associated with changes in the proviral reservoir size, we analysed the fold change in intact reservoir size from baseline in relation to the anti‐SARS‐CoV‐2‐Spike titres measured 3 months after the second and third vaccine doses, and the proportion of Spike‐specific CD4^+^ T‐cells measured 2 weeks after the second dose. We did not observe any significant correlations (Spearman, all *p* > 0.33, *r* = 0.2, −0.23, −0.03) (Figure 3b,c). Furthermore, baseline intact reservoir size was not associated with the magnitude of the vaccine‐induced Spike antibody response or the proportion of Spike‐specific CD4^+^ T‐cells following vaccination (Figure S3).

### HIV‐1 reservoir size change in correlation to immune status

**FIGURE 4:**
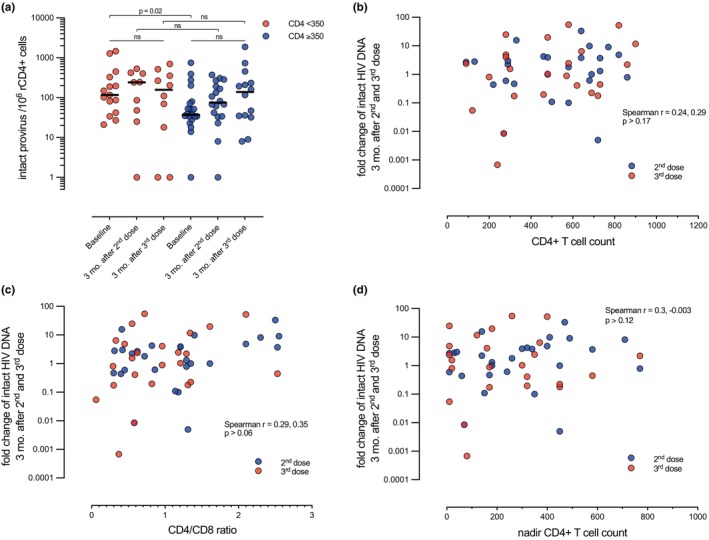
Immunological profiles and intact proviral HIV‐1 reservoir at baseline and post mRNA vaccination. (a) Intact HIV‐1 reservoir (copies/million resting CD4^+^ T‐cells) dynamics in INR (CD4 < 350 cells/mm^3^, *n* = 15) and IR (CD≥350 cells/mm^3^, *n* = 22) at baseline and after two and three doses of vaccine. A larger intact reservoir was observed in INR at baseline (Mann–Whitney U test, *p* = 0.02). No significant within‐group changes or between‐group differences of absolute values and fold change were observed following vaccination (Wilcoxon signed‐rank and Mann–Whitney U tests, all *p* ≥ 0.25). (b–d) Correlation between fold change in intact HIV‐1 DNA from baseline (day 0) to 3 months after the second (blue) and third vaccine dose (red) and (b) CD4^+^ T‐cell count (cells/mm^3^), (c) CD4^+^/CD8^+^ ratio and (d) nadir CD4^+^ T‐cell count (cells/mm^3^). No significant correlations were observed (Spearman's correlation).

To further analyse potential changes in the reservoir size, with regard to the level of immune reconstitution, the cohort was stratified into IRs (*n* = 22) (CD4^+^ T‐cell count ≥350 cells/mm^3^) and INRs (*n* = 15) (CD4^+^ T‐cell count <350 cells/mm^3^). Likewise, we could not observe any significant changes in the intact reservoir size after two and three doses of vaccine within the groups (Wilcoxon, *p* > 0.26) (Figure 4a). Furthermore, there were no significant group differences in intact reservoir size following vaccination between INRs and IRs (Mann–Whitney, *p* > 0.30) and no significant differences in fold change of intact reservoir size from baseline (Mann–Whitney, *p* > 0.25) (Figure 4a).

Additionally, the size of the intact and defective HIV‐1 reservoir did not differ significantly between males and females at baseline or during follow‐up (median 76 vs. 52 copies/million rCD4^+^ T‐cells, *p* = 0.47; Figure S1). Although males displayed a numerically larger intact reservoir throughout follow‐up, this difference was not statistically significant. Likewise, no significant differences in fold change of reservoir size from baseline were observed between sexes (Mann–Whitney *U* test, *p* > 0.18). Furthermore, we observed no association between viral suppression or transient low‐level viraemia (HIV‐1 RNA ≥20 copies/mL) and the size of the intact reservoir at baseline or after two and three vaccine doses, supporting the absence of reservoir perturbation in resting CD4^+^ T‐cells following vaccination (Figure S2).

Notably, a significantly smaller intact proviral reservoir was observed at baseline in IRs compared with INRs (37 copies/million rCD4^+^ T‐cells, IQR 33–74 vs. 117 copies/million rCD4^+^ T‐cells, IQR 42–330; *p* = 0.02) (Figure 4a). Baseline intact proviral reservoir size was also negatively correlated with CD4^+^/CD8^+^ ratio, CD4^+^ T‐cell count and nadir CD4^+^ T‐cell count (Spearman, all *p* < 0.02, *r* = −0.54, −0.43, −0.37) (Figure S4A–C).

As individuals in our cohort with lower CD4^+^ T‐cell counts, CD4^+^/CD8^+^ ratios and nadir CD4^+^ T‐cell counts had larger intact proviral reservoirs, we investigated whether baseline immune status was associated with greater changes in reservoir size following vaccination. No correlation was observed between fold change in intact reservoir size from baseline to 3 months after the second or third vaccine dose and baseline CD4^+^ T‐cell count, CD4^+^/CD8^+^ ratio and nadir CD4^+^ T‐cell count (Figure 4b–d). These findings provide additional evidence that SARS‐CoV‐2 vaccination does not perturb HIV‐1 reservoir size in people with HIV regardless of immune status or degree of immune reconstitution.

## DISCUSSION

Our study demonstrates that three doses of SARS‐CoV‐2 mRNA vaccines administered over the course of 1 year did not result in increased plasma HIV‐1 VL in people with HIV. Moreover, no significant alterations were observed in the intact, defective or total HIV‐1 reservoir size following vaccination. The magnitude of the vaccine‐induced immune response, including both Spike antibody levels and Spike‐specific T‐cell responses, was not associated with HIV‐1 viraemia or changes in the HIV‐1 reservoir size. In addition, we did not observe any significant changes in VL or HIV‐1 reservoir in INRs. Together, these findings provide reassuring evidence that mRNA vaccination does not perturb HIV persistence in people with HIV on stable ART, regardless of their immune reconstitution status.

Our findings are consistent with previous studies reporting no significant changes in the HIV‐1 reservoir or inducible viraemia following vaccination. However, our study provides valuable additional insight in the effects of vaccination in INRs, individuals with poor immune reconstitution. A study of 35 people with HIV observed a temporary increase in Nef‐specific CD8^+^ T‐cells after the first dose of mRNA vaccine, linked to immune recognition of reactivated reservoir cells, yet found no significant changes in reservoir size (*n* = 11) or plasma viraemia assessment [25]. Notably, only three INRs were included in the reservoir analysis. Another study including 23 male participants with high CD4^+^ T‐cell levels (median > 700) found no changes in reservoir size following two vaccine doses with varying follow‐up times [26]. A third study of 68 people with HIV, all males aged 55 and older, reported a small but statistically insignificant increase in VL after the second vaccine dose without clear evidence of changes in reservoir size (*n* = 41). Notably, many participants received the viral‐vectored ChAdOx1 vaccine [32]. Finally, a study involving 62 participants (94% males, CD4 > 700) receiving two doses of BNT162b2 or mRNA‐1273 found no significant changes in the intact HIV‐1 reservoir size or VL [27]. Together, these findings align with our results but are largely derived from cohorts with predominantly male participants and high CD4^+^ T‐cell counts. In contrast, our study included individuals with varying degrees of immune reconstitution, including INRs, and assessed reservoir dynamics following three vaccine doses over 1 year.

Participants in our study had an overall robust immune response to vaccination with anti‐SARS‐CoV‐2‐Spike antibody titres comparable to those of controls [4]. Here, we also show that there was no correlation between the intact reservoir size and the magnitude of the vaccine‐induced immune response, including both the antibody and spike‐specific T‐cell responses. At baseline, a proportion of the participants had transient low‐level detectable viraemia (≥20 copies/ml), consistent with viral blips. All individuals re‐suppressed to within 1 month and had no history of persistent low‐level viraemia. Detectable viraemia at baseline was not associated with intact reservoir size or vaccine‐induced immune responses, and no longitudinal relationships between transient viraemia and changes in intact proviral DNA were observed. Although viral blips have been associated with slower reservoir decay in some cohort studies [33], the impact of transient low‐level viraemia on the intact HIV reservoir remains uncertain. In our settings, these transient viral blips did not influence intact reservoir size in resting CD4+ T‐cells.

We observed a significantly smaller intact proviral reservoir in immunological responders (IRs) compared with INRs at baseline. Although a similar numerical trend persisted during follow‐up, it did not reach statistical significance at later time points, likely due to the limited sample size. Importantly, no significant increases in intact reservoir size within groups or differences in fold change between IR and INR were observed following vaccination, indicating no evidence of vaccine‐induced perturbation of the HIV‐1 reservoir even in individuals with incomplete immune reconstitution. We also observed a strong association between immune status and the size of the intact proviral reservoir at baseline. CD4^+^ T‐cell count, CD4^+^/CD8^+^ ratio and nadir CD4^+^ T‐cell count were all negatively correlated with reservoir size, with the strongest association seen for the CD4^+^/CD8^+^ ratio. Our results are in line with previous studies that have shown similar relationships [21, 22, 34, 35]. This finding emphasizes the importance of immune reconstitution, highlighting that immune recovery is linked to viral persistence and the size of the reservoir. Since lower nadir CD4^+^ levels were associated with a larger reservoir, early diagnosis of HIV and the timing of ART are crucial for shaping the size of the persistent proviral reservoir and may impact future outcomes of post‐treatment control and cure strategies. Although we observed a larger intact reservoir in INRs, we did not demonstrate a perturbation in the reservoir following vaccination or a correlation with the response to vaccination in this group. Based on this evidence, we can conclude that mRNA vaccination is safe for people with HIV regardless of their immune status.

Our diverse cohort included over 40% females, allowing us to assess potential sex‐related differences in HIV‐1 reservoir size and its dynamics following vaccination. We found no significant differences between males and females in reservoir size or longitudinal changes during follow‐up. Although females displayed a slightly smaller median reservoir size throughout the study period, this difference was not statistically significant. These findings do not contradict previous studies suggesting a smaller inducible HIV reservoir in females [36, 37].

Investigating whether mRNA vaccinations influence the HIV‐1 reservoir or induce viraemia is essential for addressing concerns about its potential impact on viral persistence and immune responses in people with HIV. In an era where vaccine hesitancy remains a challenge, particularly among vulnerable populations, studies such as ours provide important evidence‐based reassurance regarding vaccine safety. Moreover, studying reservoir dynamics in response to immunological interventions provides valuable insights into the mechanisms of HIV‐1 persistence. A deeper understanding of these processes is crucial for developing targeted strategies to reduce or eliminate the viral reservoir, thereby advancing efforts towards an HIV cure.

We acknowledge potential limitations of our study. The relatively small sample size reduces statistical power, which may limit the generalizability of our findings to the whole HIV community. As the HIV reservoir mainly resides in lymphoid tissues, the analysis of the reservoir in blood may not capture true changes occurring in the tissue reservoir. However, our study was conducted within a clinical trial, following a standardized protocol and defined sampling points, ensuring consistency. The consistent follow‐up and extent of 1 year following three doses of vaccine strengthen our findings, since immune responses and the HIV reservoir evolve over time. Furthermore, the diversity of our study population, encompassing both male and female people with HIV, as well as individuals with varying immune status, enhances the relevance and applicability of our findings.

In summary, we demonstrate that three doses of SARS‐CoV‐2 mRNA vaccine over 1 year did not induce HIV‐1 viraemia or significantly alter the size of the intact HIV‐1 reservoir in people with HIV regardless of immune status. Together with previous studies, our results provide further reassurance that mRNA vaccination does not perturb HIV persistence. Moreover, the observed association between impaired immune status and a larger reservoir highlights the critical importance of early diagnosis and initiation of ART for limiting the HIV reservoir, with potential implications for future post‐treatment control and HIV cure strategies.

## AUTHOR CONTRIBUTIONS

P.N., P.S. and O.K. conceptualized the study. S.A. was PI of the COVAXID clinical trial. O.K., J.V., S.A. and P.N. recruited patients. P.S., B.B.J., M.B. and A.S. extracted and analysed samples. O.K. and P.S. collected and analysed data. P.N., P.S. and A.S. supervised and made intellectual input. O.K. wrote the original draft. All authors reviewed, revised and approved the final manuscript.

## FUNDING INFORMATION

This work was supported by the Swedish Research Council [2024‐06337 to P.N.], Stockholm County Council Swedish [SLL‐KI FoUI‐995225 and CIMED FoUI‐1002100 to P.N.], the Physicians Against AIDS Research Foundation [FOa2022‐0001 and FOa2023‐0003 to B.B.J.] and the Karolinska Institutet‐KID funding [2021‐00552 to O.K.].

## CONFLICT OF INTEREST STATEMENT

The authors declare no conflicts of interest.

## Supporting information


**Figure S1.** HIV‐1 reservoir dynamics in females and males after of SARS‐CoV‐2 mRNA vaccinations. Intact HIV‐1 reservoir size (copies/million resting CD4^+^ T‐cells) in females (*n* = 15) and males (*n* = 22) at baseline and throughout follow‐up, within‐ and between‐group comparisons of absolute values and fold change (Wilcoxon signed‐rank test and Mann–Whitney *U* test, all *p* ≥ 0.18).

**Figure S2.** Viral suppression and viraemia in relation to intact HIV‐1 reservoir size. Intact HIV‐1 reservoir size (copies/million resting CD4^+^ T‐cells) in people with HIV with viral suppression (HIV‐1 RNA <20 copies/ml, LLOQ) (blue) and viraemia (HIV‐1 RNA ≥20 copies/ml) (red). Baseline, pre‐vaccine, HIV‐1 RNA <20; 52 (34–117) (*n* = 27), versus HIV‐1 RNA ≥20; 126 (31–612) (*n* = 10) (Mann–Whitney U test). Three months after second dose, HIV‐1 RNA <20; 97 (40–251) (*n* = 22), versus HIV‐1 RNA ≥20; 48 (29–412) (*n* = 5). Three months after third dose, HIV‐1 RNA <20; 117 (34–486) (*n* = 17), versus HIV‐1 RNA <20; 115 (14–289) (*n* = 6).

**Figure S3.** Intact proviral HIV‐1 reservoir size at baseline in relation to vaccine‐induced immune responses. Intact proviral reservoir at baseline in correlation to (A) vaccine‐induced SARS‐CoV‐2‐Spike antibody titres after two (blue) and three (red) doses of vaccine and (B) percentage of Spike‐specific CD4^+^ T cells 2 weeks after two doses (Spearman's correlation). (C) SARS‐CoV‐2 Spike antibody titres stratified by baseline intact reservoir size using the cohort median as cut‐off, high (>53 intact DNA copies/million rCD4^+^ T cells, *n* = 17) and low (≤53 intact DNA copies/million rCD4^+^ T cells, *n* = 20) (Mann–Whitney *U* test).

**Figure S4.** Immunological profiles and intact proviral HIV‐1 reservoir size at baseline. Correlation between intact proviral HIV‐1 DNA measured by IPDA (copies per million resting CD4^+^ T cells) at baseline (day 0) and immunological parameters. Intact reservoir size showed negative correlations with (A) CD4^+^ T‐cell count, (B) nadir CD4^+^ T‐cell count and (C) CD4^+^/CD8^+^ ratio (Spearman's correlation).

## Data Availability

The data that support the findings of this study are available from the corresponding author upon reasonable request.
