# Supplementary figures and images for: HIV‐1 viral load and reservoir size remain stable following SARS‐CoV‐2 mRNA vaccination in people with HIV
Source: HIV Med. 2026 Apr 5;27(7):1120–31. doi: 10.1111/hiv.70235 (PMC13340954; doi:10.1111/hiv.70235)

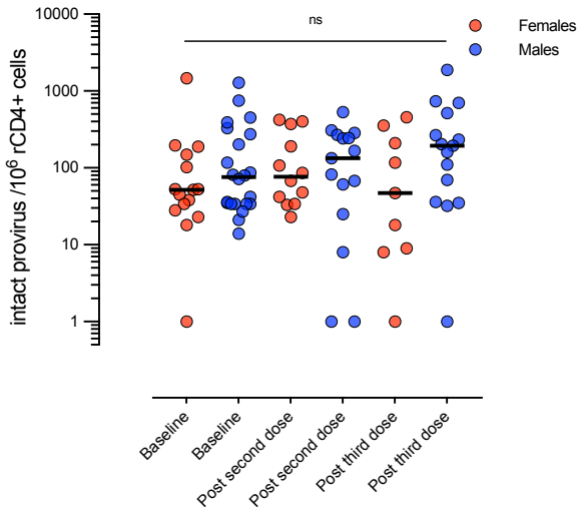

Supplement: Supplementary file 1 — Figure S1. HIV‐1 reservoir dynamics in females and males after of SARS‐CoV‐2 mRNA vaccinations. Intact HIV‐1 reservoir size (copies/million resting CD4+ T‐cells) in females (n = 15) and males (n = 22) at baseline and throughout follow‐up, within‐ and between‐group comparisons of absolute values and fold change (Wilcoxon signed‐rank test and Mann–Whitney U test, all p ≥ 0.18). [file HIV-27-1120-s003.pdf]

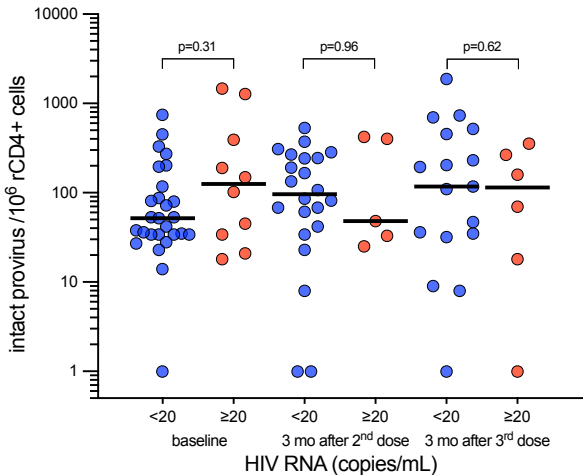

Supplement: Supplementary file 2 — Figure S2. Viral suppression and viraemia in relation to intact HIV‐1 reservoir size. Intact HIV‐1 reservoir size (copies/million resting CD4+ T‐cells) in people with HIV with viral suppression (HIV‐1 RNA <20 copies/ml, LLOQ) (blue) and viraemia (HIV‐1 RNA ≥20 copies/ml) (red). Baseline, pre‐vaccine, HIV‐1 RNA <20; 52 (34–117) (n = 27), versus HIV‐1 RNA ≥20; 126 (31–612) (n = 10) (Mann–Whitney U test). Three months after second dose, HIV‐1 RNA <20; 97 (40–251) (n = 22), versus HIV‐1 RNA ≥20; 48 (29–412) (n = 5). Three months after third dose, HIV‐1 RNA <20; 117 (34–486) (n = 17), versus HIV‐1 RNA <20; 115 (14–289) (n = 6). [file HIV-27-1120-s001.pdf]

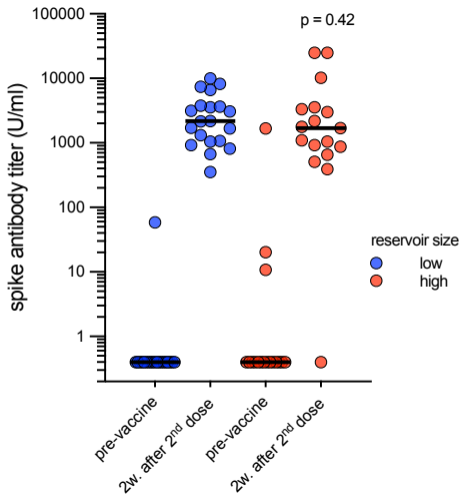

Supplement: Supplementary file 3 — Figure S3. Intact proviral HIV‐1 reservoir size at baseline in relation to vaccine‐induced immune responses. Intact proviral reservoir at baseline in correlation to (A) vaccine‐induced SARS‐CoV‐2‐Spike antibody titres after two (blue) and three (red) doses of vaccine and (B) percentage of Spike‐specific CD4+ T cells 2 weeks after two doses (Spearman's correlation). (C) SARS‐CoV‐2 Spike antibody titres stratified by baseline intact reservoir size using the cohort median as cut‐off, high (>53 intact DNA copies/million rCD4+ T cells, n = 17) and low (≤53 intact DNA copies/million rCD4+ T cells, n = 20) (Mann–Whitney U test). [file HIV-27-1120-s004.zip › hiv70235-sup-0006-FigureS3@supp fig 3c.pdf]

intact provirus /  $10^6$  rCD4+ cells

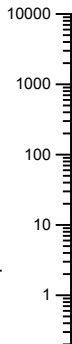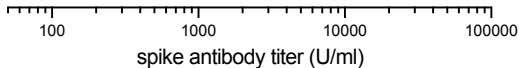

Spearman  $r = 0.27, -0.15$   
 $p > 0.27$

● 2<sup>nd</sup> dose  
● 3<sup>rd</sup> dose

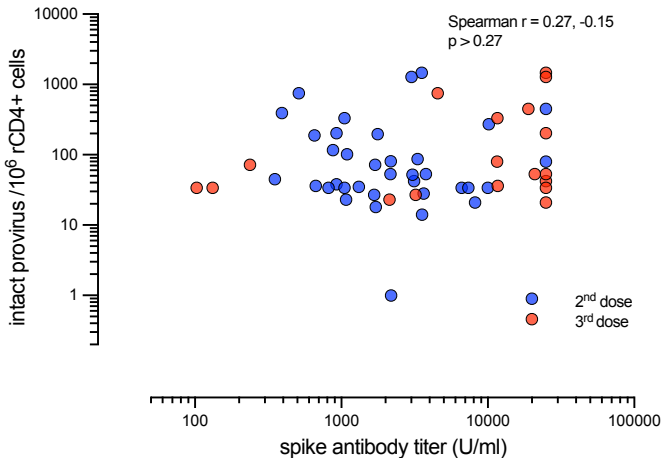

Supplement: Supplementary file 3 — Figure S3. Intact proviral HIV‐1 reservoir size at baseline in relation to vaccine‐induced immune responses. Intact proviral reservoir at baseline in correlation to (A) vaccine‐induced SARS‐CoV‐2‐Spike antibody titres after two (blue) and three (red) doses of vaccine and (B) percentage of Spike‐specific CD4+ T cells 2 weeks after two doses (Spearman's correlation). (C) SARS‐CoV‐2 Spike antibody titres stratified by baseline intact reservoir size using the cohort median as cut‐off, high (>53 intact DNA copies/million rCD4+ T cells, n = 17) and low (≤53 intact DNA copies/million rCD4+ T cells, n = 20) (Mann–Whitney U test). [file HIV-27-1120-s004.zip › hiv70235-sup-0004-FigureS3@supp fig 3a.pdf]

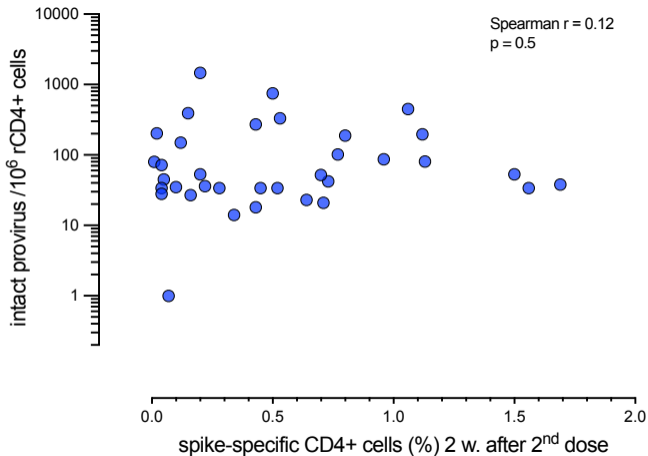

Supplement: Supplementary file 3 — Figure S3. Intact proviral HIV‐1 reservoir size at baseline in relation to vaccine‐induced immune responses. Intact proviral reservoir at baseline in correlation to (A) vaccine‐induced SARS‐CoV‐2‐Spike antibody titres after two (blue) and three (red) doses of vaccine and (B) percentage of Spike‐specific CD4+ T cells 2 weeks after two doses (Spearman's correlation). (C) SARS‐CoV‐2 Spike antibody titres stratified by baseline intact reservoir size using the cohort median as cut‐off, high (>53 intact DNA copies/million rCD4+ T cells, n = 17) and low (≤53 intact DNA copies/million rCD4+ T cells, n = 20) (Mann–Whitney U test). [file HIV-27-1120-s004.zip › hiv70235-sup-0005-FigureS3@supp fig 3b.pdf]

intact provirus /  $10^6$  rCD4+ cells

Spearman  $r = -0.43$   
 $p = 0.008$

CD4+ T cell count at baseline

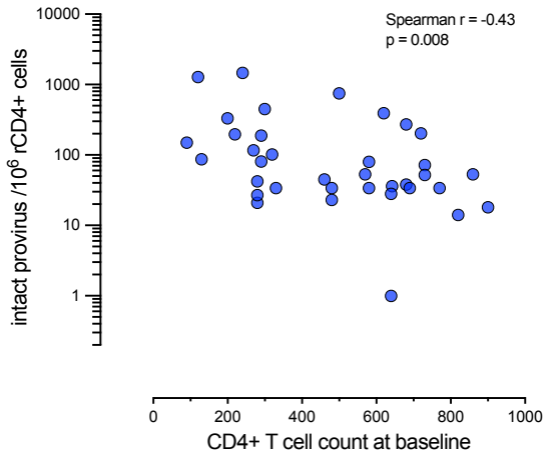

Supplement: Supplementary file 4 — Figure S4. Immunological profiles and intact proviral HIV‐1 reservoir size at baseline. Correlation between intact proviral HIV‐1 DNA measured by IPDA (copies per million resting CD4+ T cells) at baseline (day 0) and immunological parameters. Intact reservoir size showed negative correlations with (A) CD4+ T‐cell count, (B) nadir CD4+ T‐cell count and (C) CD4+/CD8+ ratio (Spearman's correlation). [file HIV-27-1120-s002.zip › hiv70235-sup-0007-FigureS4@supp fig 4a.pdf]

intact provirus /  $10^6$  rCD4+ cells

Spearman  $r = -0.37$   
 $p = 0.02$

nadir CD4+ T cell count

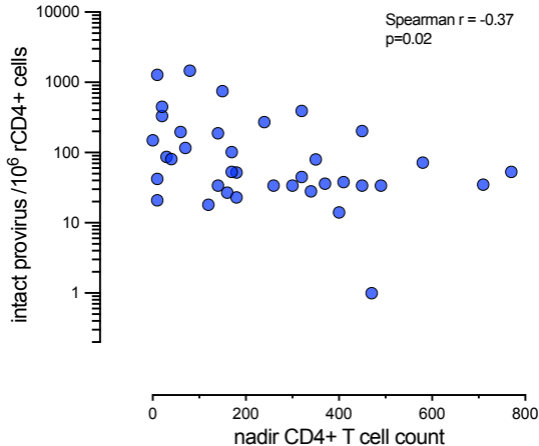

Supplement: Supplementary file 4 — Figure S4. Immunological profiles and intact proviral HIV‐1 reservoir size at baseline. Correlation between intact proviral HIV‐1 DNA measured by IPDA (copies per million resting CD4+ T cells) at baseline (day 0) and immunological parameters. Intact reservoir size showed negative correlations with (A) CD4+ T‐cell count, (B) nadir CD4+ T‐cell count and (C) CD4+/CD8+ ratio (Spearman's correlation). [file HIV-27-1120-s002.zip › hiv70235-sup-0008-FigureS4@supp fig 4b.pdf]

intact provirus /10<sup>6</sup> rCD4+ cells

Spearman  $r = -0.54$   
 $p = 0.0006$

CD4/CD8 ratio at baseline

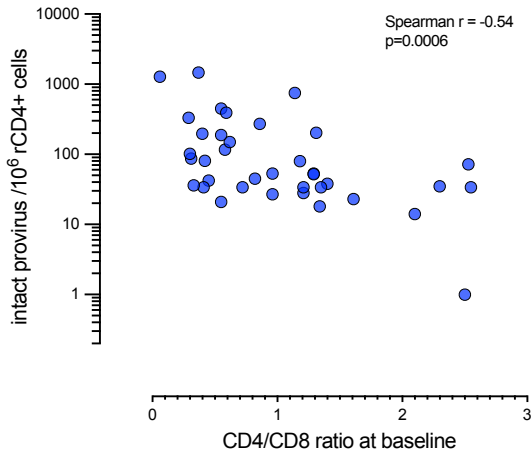

Supplement: Supplementary file 4 — Figure S4. Immunological profiles and intact proviral HIV‐1 reservoir size at baseline. Correlation between intact proviral HIV‐1 DNA measured by IPDA (copies per million resting CD4+ T cells) at baseline (day 0) and immunological parameters. Intact reservoir size showed negative correlations with (A) CD4+ T‐cell count, (B) nadir CD4+ T‐cell count and (C) CD4+/CD8+ ratio (Spearman's correlation). [file HIV-27-1120-s002.zip › hiv70235-sup-0009-FigureS4@supp fig 4c.pdf]
